# Acupuncture at Zusanli (ST36) for the benefits of sepsis–laboratory evidence for “preventive treatment of disease” and “holistic concept” in the protective effects of acupuncture: a literature review of rodent studies

**DOI:** 10.3389/fneur.2025.1645906

**Published:** 2025-09-22

**Authors:** Lin Zeng, Jiangtian Yan

**Affiliations:** ^1^LiShizhen College of Traditional Chinese Medicine, Huanggang Normal University, Huanggang, China; ^2^Hubei Key Laboratory of Germplasm Improvement and Utilization of Dabie Shan Dao-di Herbs, Huanggang Normal University, Huanggang, China; ^3^LiShizhen Culture and Industry Research Center of Traditional Chinese Medicine, Huanggang, China

**Keywords:** acupuncture, Zusanli (ST36), sepsis, neuro-endocrine-immune network, exosome, review

## Abstract

The objective of this study was to explore the holistic anti-inflammatory mechanisms of acupuncture at ST36 in sepsis-related multi-organ damage. Current research indicates that ST36 stimulation neuro-endocrine-immune network and serum exosome, which exhibit anti-inflammatory properties in sepsis or normal mice. However, critical gaps persist: Firstly, there is a necessity for further exploration of neural circuit mechanisms, given that the effects of acupuncture involve multiple interdependent pathways [e.g., sympathetic nervous system, vagus nervous system (cholinergic anti-inflammatory pathway and vagal- splanchnic nerve axis)], which underscores the importance of studies on context-specific neural conduction conditions. Secondly, there is a lack of clarity regarding exosome dynamics, including their production mechanisms, cellular origins, and optimal therapeutic targets. The last, while the majority of studies have focused on post-inflammatory regulation, emerging evidence suggests the potential for preventive applications, such as the observation that low-intensity ST36 electroacupuncture activates disease-independent anti-inflammatory pathways. Future research must integrate neural circuit complexity, exosome biology, and preventive applications to advance acupuncture’s translational utility in sepsis and systemic inflammation.

## 1 Introduction

The third international consensus definition of sepsis, published in JAMA in 2016, defines sepsis as life-threatening organ dysfunction caused by a dysregulated host response to infection (1). Advances in modern medicine have led to a decline in the overall mortality rate for patients admitted to hospitals with severe sepsis. Nevertheless, the mortality rate (>30%) remains unacceptably high (2). The most prevalent origins of sepsis are respiratory, gastrointestinal, genitourinary, and skin and soft tissue infections (3). Among these, pneumonia is the most prevalent cause of sepsis (3). The primary mechanism that precipitates sepsis is inflammation (4), which generally manifests in two phases: a pro-inflammatory phase, termed systemic inflammatory response syndrome (SIRS), and an anti-inflammatory phase, designated as compensatory anti-inflammatory response syndrome (CARS) (5). Furthermore, depending on the severity of the infection that caused the sepsis, the immune system may never fully recover, resulting in long-term immune dysfunction (6). The potential for therapeutic interventions to mitigate complications arising from sepsis is predicated on the restoration of disrupted adaptive and innate immune responses, or the reduction of pro-inflammatory mediator levels (4).

In the domain of traditional Chinese medicine (TCM), acupuncture stands as a distinctive and significant technique for addressing and preventing diseases. In the contemporary era, acupuncture has gained global recognition and acceptance, having been endorsed by prominent health authorities such as the World Health Organization (WHO) and the National Institutes of Health (NIH). A mounting body of evidence supports the efficacy of acupuncture in anti-inflammatory effects (7, 8). Moreover, a substantial corpus of research substantiates the immune-regulating effects of acupuncture (9). Electroacupuncture (EA) constitutes a novel technique for treating diseases that involves the use of acupuncture needles in conjunction with modern technology. It is also extensively employed in fundamental research to investigate the mechanisms of acupuncture in disease management due to its capacity to adjust the parameters of the stimulation current. To ensure clarity and consistency in the subsequent descriptions in this paper, the term “acupuncture” is used uniformly. Basic research on the anti-inflammatory or immune-modulating effects of acupuncture on sepsis accurately reflects the practical significance and important supporting evidence of two key concepts in traditional Chinese medicine, namely ‘preventive treatment of diseases’ and the ‘holistic concept’. These principles find expression in the practice of acupuncture, which is employed for the prevention and treatment of diseases. With regard to the “preventive treatment of diseases “, recent research has demonstrated the efficacy of acupuncture in preventing and treating sepsis (10–12), furthermore, studies on other ailments have also yielded promising results (13, 14). A substantial body of laboratory research has investigated the anti-inflammatory and immune-modulating properties of acupuncture in relation to multi-organ damage caused by sepsis, which is a direct manifestation of the ‘holistic concept’.

A review of the extant literature on the relationship between acupuncture and sepsis reveals that, on the one hand, previous studies have provided only a broad summary of the molecular mechanisms by which acupuncture alleviates sepsis through immune regulation and organ protection. For instance, Yang et al. and Wang et al. conducted exhaustive reviews on the molecular mechanisms underlying the relationship between acupuncture and its ability to alleviate sepsis (11, 12). Moreover, there are studies that summarize the neural circuit mechanisms related to acupuncture’s regulation of sepsis immunity from the perspective of neural transmission. For instance, Pan et al. and Zhang et al. conducted exhaustive reviews on the regulatory effects of peripheral nerve stimulation induced by acupuncture on sepsis immunity (10, 15). However, it is the contention of the present study that research on sepsis, a clinically critical condition, should focus on prevention and protection, in a manner analogous to research on cerebral ischaemia-reperfusion injury (16, 17). This finding is consistent with the growing body of research employing acupuncture as a preconditioning/pretreatment modality in sepsis-related studies. Accordingly, the present study has identified the acupoint ST36 (8) as the primary subject for investigation on the basis of its established anti-inflammatory and immune-modulating properties. The importance of ST36 acupoint can be considered from three perspectives. Firstly, it is a well-established acupoint for health preservation and wellness, with numerous related basic research studies to support its efficacy. Secondly, a single acupoint, especially one located on the limbs, is more conducive to studying neural circuit transmission. Thirdly, applying different stimulation intensities to ST36 results in distinct pathways through which its effects are mediated. Moreover, recent literature on the anti-inflammatory effects of serum exosome from normal mice following acupuncture at ST36 (18, 19) has been added. The primary objective of this study is to elucidate the mechanisms by which the pre-acupuncture effects at ST36 are transmitted and manifested. In addition, the study proposes a series of recommendations for future basic research on acupuncture preconditioning/pretreatment.

## 2 Sources and selection criteria

A comprehensive search was conducted on PubMed, with a focus on original paper publications from the database’s inception until June 1, 2025. Keywords included “acupuncture” or “electroacupuncture,” and “mouse” or “rat” or “mice” or “rats,” and “sepsis.” The inclusion criteria for the study were as follows: Articles had to be original research papers that utilized lipopolysaccharide (LPS) or cecum ligation and perforation surgery (CLP) to induce sepsis in an *in vivo* experimental model (rats or mice). Additionally, the studies had to exclusively focus on acupuncture at the ST36 acupoint. A comprehensive search of the extant literature yielded a total of 21 articles (Table 1) (18–38). After undergoing thorough evaluation, the information provided in the subsequent studies was described and deliberated upon.

**Table 1:** Effects of acupuncture on the sepsis.

Refs.	Sepsis’s induction method	Intervention methods	Acupoints	Acupuncture parameters	Which organ was damaged?	Test sites	Biochemical measurements	How to block the effect?
Huang et al. (20)	LPS (20 mg/kg) intraperitoneal injection, Sprague–Dawley rats.	Manual acupuncture pretreatment	ST36	None.	Lung	Blood, Lung	Blood: NO ↓. Lung: NO ↓, MPO ↓, iNOS ↓.	None.
Huang et al. (21)	LPS (20 mg/kg) intraperitoneal injection, Sprague–Dawley rats.	Manual acupuncture pretreatment	ST36	twirled and twisted (> 360°) at a frequency of 60 turns/min throughout the 30 min.	Kidney, Liver	Plasma, Kidney, Liver	Plasma: BUN ↓, Cr ↓, AST -, ALT -, Total bilirubin -. Kidney: NO ↓, iNOS ↓. Liver: NO -, iNOS -.	None.
Shi et al. (22)	CLP, Sprague–Dawley rats.	EA	ST36	2 mA, 2/100 Hz, 1 h.	Liver	Plasma, Liver	Plasma: ALT ↓. Liver: MDA ↓, XOD ↓.	Subdiaphragmatic vagotomy.
Wang et al. (23)	CLP, Sprague–Dawley rats.	EA	ST36	2 ~ 3 mA, 2 ~ 100 Hz, 30 min.	Brain	Plasma, Brain	Plasma: NSE ↓. Brain: TNF-*α* ↓, IL-6 ↓.	None.
Villegas-Bastida et al. (24)	CLP, Wistar rats.	EA	ST36	40 mA, 30 Hz, 50 μs, 20 min.	Liver, Kidney, Lung	Serum, Spleen	Serum: TNF ↓, IL-6 ↓, Nitrite ↓, HMGB1 ↓. Spleen: NF-κB p65 ↓.	Subdiaphragmatic vagotomy.
Wu et al. (25)	CLP, Wistar rats.	EA	ST36	3 mA, 2 ~ 100 Hz, 30 min.	Jejunum	Serum, Jejunum	Serum: HMGB1 ↓, Ghrelin ↑. Jejunum: HMGB1 ↓, Ghrelin ↑.	Inject Ghrelin-receptor blocker [D-Arg ^1^, D-Phe ^5^, D-Trp ^7.9^, Leu ^11^] substance P (700 nmol/kg, diluted with 0.9% NaCl to obtain a solution of 1 mL) at the tail vein.
Wu et al. (26)	CLP, Sprague–Dawley rats.	EA	ST36	3 mA, 2 ~ 100 Hz, 30 min.	Intestine	Serum, Intestine	Serum: Ghrelin ↑, TNF-α ↓, HMGB1 ↓. Intestine: Ghrelin ↑, Ghrelin-receptor ↑, HMGB1 ↓, MPO ↓, DAO ↑.	Inject Ghrelin-receptor [D-Arg ^1^, D-Phe ^5^, D-Trp ^7.9^, Leu ^11^] blocker substance P (700 ng/kg, diluted with 0.9% NaCl to obtain a solution of 1 ml) at the tail vein.
Zhang et al. (27)	CLP, Wistar rats.	EA	ST36	3 mA, 4/50 Hz (alternating every 6 s) and pulse width 0.5 ms, 30 min.	Ileum	Serum, Ileum	Serum: D-lactate ↓. Ileum: Occludin ↑.	None.
Zhang et al. (28)	CLP, Sprague–Dawley rats.	EA	ST36	2 mA, 2-100 Hz, 1 h.	Heart	Plasma, Cardiac muscle	Plasma: CK-MB ↓. Cardiac muscle: TNF-α ↓, NO ↓, MPO ↓.	Abdominal vagotomy.
Harpin et al. (29)	Intraperitoneal administration of live *Eschericia coli* bacteria *ATCC 25922*, Wistar rats.	EA pretreatment	ST36	2 Hz, 30 min.	Kidney	Blood	Blood: Urea ↓, Creatinine ↓.	None.
Liu et al. (30)	LPS intraperitoneal injection, 8 mg/kg for C57BL/6 J mice and 12 mg/kg for mix genetic background mice.	EA pretreatment	ST36	0.5 mA.	Systemic Inflammation	Serum, Spleen, Brain (Dorsal Motor Nuclei of the Vagus)	Serum: NA ↑, A ↑, DA ↑, TNF-α ↓, IL-6 ↓. Spleen -. Brain: c-Fos ↑.	NPY^DBH^ (peri.)-Abl mice, Subdiaphragmatic Vagotomy.
Xie et al. (31)	CLP, Sprague–Dawley rats.	EA	ST36	2 V (load voltage), 3 Hz, 15 min.	Intestine	Serum, Intestine	Serum: TNF-α ↓, IL-10 ↓, D-LA ↓, DAO ↓. Intestine: CD3 + CD4 + (%) ↑, CD3 + CD8 + (%) ↓, CD3 + CD4+/CD3 + CD8 + ↑, Treg (%) ↑, Th17 (%) ↓, Treg/Th17 ↑.	Postsplenectomy (SPX), GTS21(a specific agonist of *α*7nAchR, 4 mg/kg/d).
Liu et al. (32)	LPS intraperitoneal injection, 8 mg kg^−1^ for C57BL/6 J mice and 12 mg kg ^−1^ for mice with a mixed genetic background.	EA	ST36	0.3 mA.	Systemic Inflammation	Serum, Brain (Dorsal Motor Nuclei of the Vagus)	Serum: NA ↑, A ↑, DA ↑, TNF-α ↓, IL-6 ↓. Dorsal Motor Nuclei of the Vagus (DMV): Fos ↑.	*Prokr2^Adv^-DTR* mice.
Zhang et al. (33)	LPS (5 mg/kg) intraperitoneal injection, C57BL/6 mice.	EA	ST36	0.5 mA, 4/20 Hz, 20 min.	Lung	Lung	Lung: GPX4 ↑, SLC7A11 ↑, FTH1 ↑, Iron ↓, GSH ↑, MDA ↓, TNF-α ↓, IL-6 ↓, ROS ↓, α7nAchR ↑.	Methylaconitine (MLA, 5 mg/kg), a selective a7nAchR antagonist, Sciatic nerve transection (SCT), Left cervical vagotomy (LCV).
Lv et al. (34)	LPS (4 mg/kg) intraperitoneal injection, BALB/c mice.	EA pretreatment	ST36	0.1 mA, 10 Hz, 30 min.	Intestine, Lung	Serum, Spleen, Intestine, Lung	Serum: TNF-α ↓, IL-1β ↓, IL-4 ↓, IL-5 ↓, IL-6 ↓, IL-9 ↓, IL-10 ↓, IL-17A ↓, IFN-γ ↓, eotaxin ↓, MIP-1β ↓, KC ↓. Spleen: T lymphocyte apoptosis (%) ↓, cleaved caspase-1 (Asp296) ↓.	Nude mice.
Lou et al. (35)	CLP, Sprague–Dawley rats.	EA pretreatment	ST36	2 mA, 2 Hz, 30 min.	Small Intestine	Liver, Spleen, Small Intestine	Small Intestine: CD4 + (%) ↑, CD8 + (%) ↑, IL-4 ↑, sIgA ↑, Bcl-2 ↑, Bax ↓.	None.
Zhan et al. (36)	LPS (10 mg/mL) intraperitoneal injection, Institute of *Cancer* Research (ICR) mice.	EA pretreatment	ST36	2.5 mA, 2-100 Hz, 30 min.	Colon	Serum, Colon	Serum: LDH ↓, IL-1β ↓, IL-6 ↓, TNF-α ↓, IL-10 ↑. Colon: caspase 3 ↓, cleaved caspase 3 ↓, Bcl-2 ↑, Bax ↓, TLR4 ↓, MyD88 ↓, p–NF–κB/NF-κB ↓.	None.
Liu et al. (37)	LPS (5 mg/kg) intraperitoneal injection, Sprague–Dawley rats.	EA pretreatment	ST36	1 ~ 3 mA, 4/20 Hz, 30 min.	Lung	BALF, Lung	BALF: IL-1β ↓, TNF-α ↓, Ang (1–7) ↑. Lung: ACE2 ↑, MasR ↑.	None.
Zhang et al. (18)	LPS (12 mg/kg, 24 mg/kg) intraperitoneal injection, C57BL/6 J mice.	EA pretreatment, Acu-exo pretreatment	ST36	intensity 5, 10 Hz, 15 min.	Lung	Blood, Lung	Blood: TNF-α ↓, IL-6 ↓, IL-1β ↓.	None.
Li et al. (19)	LPS (10 mg/kg) intraperitoneal injection, C57BL/6 J mice.	Acu-exo (4 mg/kg)	ST36	0.76 mA, 10 Hz, 15 min.	Lung	Serum, Lung	Serum: IL-6 ↓, IL-1β ↓, TNF-α ↓. Lung: ROS ↓, MPO ↓, SOD ↑, MDA ↓, GSH-Px ↑, IL-6 ↓, IL-1β ↓, TNF-α ↓, caspase 1 ↓, IL-1β ↓, p–NF–κB/NF-κB ↓, NLRP3 ↓.	P2X7 KO mice.
Huang et al. (38)	LPS (15 mg/kg) was administered intravenously via the tail vein, C57BL/6 mice.	EA	ST36	1 mA, 10 Hz, 15 min.	Lung	BALF, Serum, Lung	BALF: LXA4 ↑, TNF-α ↓, IL-6 ↓, IL-1β ↓, Serum: LXA4 ↑.	Bilateral cervical vagotomy, α7nAChR agonist (PNU-282987), LXA4 inhibitor WRW4.

## 3 Current methods of inducing sepsis

Animal models provide a bridge between patients and the laboratory bench (39). A hyperinflammatory state known as SIRS occurs during sepsis in both rodents and humans (40). Sepsis induced by CLP (41) and LPS-induced sepsis (42) have been observed to closely resemble the clinical presentation of human sepsis (43–46). This result is in perfect agreement with the modeling approach that has been covered in the literature included in this paper.

## 4 Acupuncture at Zusanli (ST36) for the benefits of sepsis--laboratory evidence for the “holistic concept”

### 4.1 The causes of multiple organ damage caused by sepsis

Sepsis can be defined as an inflammatory disease mediated by the host immune response (47). The two major categories of the immune system are the innate immune system and the adaptive immune system. In the early stages of sepsis, both of these systems can release numerous inflammatory cytokines in order to eliminate foreign pathogens (48). In the early stages of severe infection, the innate immune system is triggered first. Necrotic tissue and/or microorganisms release a range of destructive substances, consisting of damage-associated molecular patterns (DAMPs) and pathogen-associated molecular patterns (PAMPs), respectively. The aforementioned substances have been demonstrated to induce the rapid activation of a series of membrane receptors, designated pattern recognition receptors (PRRs), including toll-like receptors (TLRs) expressed by cells of the innate immune system. The subsequent intracellular signal transduction process is characterized by a high degree of complexity, with a multitude of complementary and/or redundant signaling pathways ultimately giving rise to the expression of genes that are involved in adaptive immunity and inflammation (47). It has been demonstrated that, as a consequence of this process, the local reaction becomes a systemic reaction, which in turn leads to widespread infection throughout the body (49). These include specific cells that are equipped with pathogen-detecting components, including endothelial cells, dendritic cells, natural killer cells, monocytes in the blood, and macrophages in tissues. Following activation, these cells persist in the production and release of substantial quantities of inflammatory mediators. Key inflammatory factors include interleukin (IL)-1β, IL-2, IL-6, tumor necrosis factor (TNF)-*α*, and chemokines such as prostaglandins, histamine, and IL-8. These molecules have been found to target vascular endothelial cells (ECs), leading to the release of nitric oxide (NO) and subsequently increasing vascular permeability (50). In this instance, IFN (Interferon)-*α*, produced by ECs, activates dendritic cells, monocytes, and natural killer (NK) cells. These cells then release cytokines (e.g., IFN-*γ*) to enhance the immune response (51). Activation of TLR-4 has been demonstrated to result in the recruitment of harmful neutrophils to the site of injury (52), followed by the exacerbation of damage caused by the large amount of reactive oxygen species (ROS) produced by these cells. The aforementioned changes, when considered in combination, have been demonstrated to result in an escalation in the severity of sepsis, ultimately leading to multiple organ failure and death (53, 54).

The activation of the innate immune system is followed by the participation of T cells and B cells of the adaptive immune system through antigen-specific responses. The activation of CD4^+^ T cells promotes their polarization into specific Th subpopulations, including Th1, Th2, and Th17 (55). Th1 cells have been identified as being essential for the expansion of memory T cells through IL-2 secretion and for the initiation of CD8^+^ T cell activation (56). Furthermore, Th1 cells primarily secrete the pro-inflammatory cytokine IFN-*γ*, which serves to further promote phagocytosis and the eradication of microorganisms (57). Conversely, Th2 cells have been observed to induce B lymphocyte category conversion through the release of IL-4 and IL-5, culminating in the subsequent release of IL-10 to mitigate inflammation (58). In summary, the dynamic equilibrium between Th1 and Th2 is imperative for the resolution of infections. Consequently, when this equilibrium is compromised, such as during sepsis, it may result in the development of secondary infections (59). Th17 cells have been shown to have a specific effect on bacterial and extracellular fungal pathogens, producing cytokines such as TNF-*α*, IL-17, and IL-22 (60). Furthermore, CD8^+^ T cells have been shown to facilitate the clearance of infections and are responsible for the production of memory CD8^+^ T cells in response to infection (56). The binding of CD8^+^ cells to homologous antigens in the presence of cytokines and co-stimulatory molecules has been shown to trigger the cytotoxic function of CD8^+^ T cells. This event leads to rapid proliferation and expansion, with the result that effector functions are conferred, including the release of TNF-*α* and IFN-*γ*, as well as cytotoxic functions (61, 62). Regulatory T cells (T_regs_) account for less than 10% of the total number of CD4^+^ T cells in lymph nodes and circulation, and play a key role in the regulation of immune cells in both steady-state and disease environments (56). T_regs_ respond to infection by suppressing excessive immune responses caused by other cells of the adaptive immune system, thereby inhibiting inflammation. Furthermore, it has been demonstrated that they maintain self-tolerance by secreting transforming growth factor (TGF)-*β* and IL-10, and target antigen presentation by dendritic cells (63). In comparison with other T cell populations, the atypical T cell subpopulation (termed ‘*γ*δ T cells’) accounts for approximately 0.5–5% of circulating cells (56). The surfaces of these cells are distinguished by the presence of unique T cell receptors (TCRs), which are comprised of γ chains and *β* chains, rather than the more traditional *α* chains and β chains. The function of these cells is to maintain immune homeostasis in lung and intestinal epithelial cells, thereby preventing pneumonia and intestinal infections, respectively. These cells have been shown to mediate protective functions against invading pathogens by releasing IL-17 and IFN-*γ* (64). B cells are pivotal to the adaptive immune response, orchestrating the production of antigen-specific antibodies against specific pathogens (56). In the context of sepsis, these processes become severely disrupted, thereby impeding the capacity of the adaptive immune system to mount an effective defensive response to infection (63). Research has demonstrated that the depletion of CD4^+^ and CD8^+^ T cells can result in lymphopenia, which, in turn, can lead to abnormal clonal expansion and an increased probability of viral reactivation in affected patients (65). Concurrently, the primary functions of B cells, encompassing antibody production and antigen presentation to T cells, are markedly impaired. The overall proportion of spleen- and tissue-specific B cells decreases, and the production of antigen-specific antibodies is impaired (66).

In essence, the body’s inability to regulate excessive inflammation can result in a multitude of symptoms associated with sepsis, including disseminated intravascular coagulation (DIC) and subsequent multiple organ dysfunction syndrome (MODS). This is characterized by inflammatory coagulation caused by abnormal platelet activation, peripheral vasodilation leading to hypotension, and subsequent inadequate renal perfusion and renal failure (67, 68).

### 4.2 Acupuncture can protect against multiple organ damage caused by sepsis

One of the fundamental principles of the “holistic concept” in Chinese medicine is the notion that the human body is considered an organic whole. The human body is composed of a number of internal organs, tissues and other organs. Each viscera, tissue and organ possesses its own unique physiological functions, and these different functions are an integral part of the overall activity of the body, which determines the unity within the body. There has been a paucity of literature specifically addressing basic experimental research focusing on the “holistic concepts” of Chinese medicine. However, sepsis can be regarded as a suitable disease model with which to explore the aforementioned “holistic concepts.” The progression of sepsis leads to the impairment of numerous organs throughout the body, including the liver, heart, lungs, kidneys, nervous system and so on (69, 70).

Acupuncture, a component of Chinese medicine that has been proven to be both green and safe, has been the focus of an increasing number of basic research studies. Concurrently, studies have identified ST36, a classical acupoint in Chinese medicine, as a promising therapeutic modality for various systemic diseases (71). The fundamental mechanism underlying its therapeutic effects involves its capacity to modulate inflammation (8). The integration of these two factors suggests that sepsis plus acupuncture can serve as a suitable combination for studying the comprehensive modulation of anti-inflammatory effects. This finding is consistent with the current body of research examining the transmission of acupuncture effects (32, 72).

**Figure 1:**
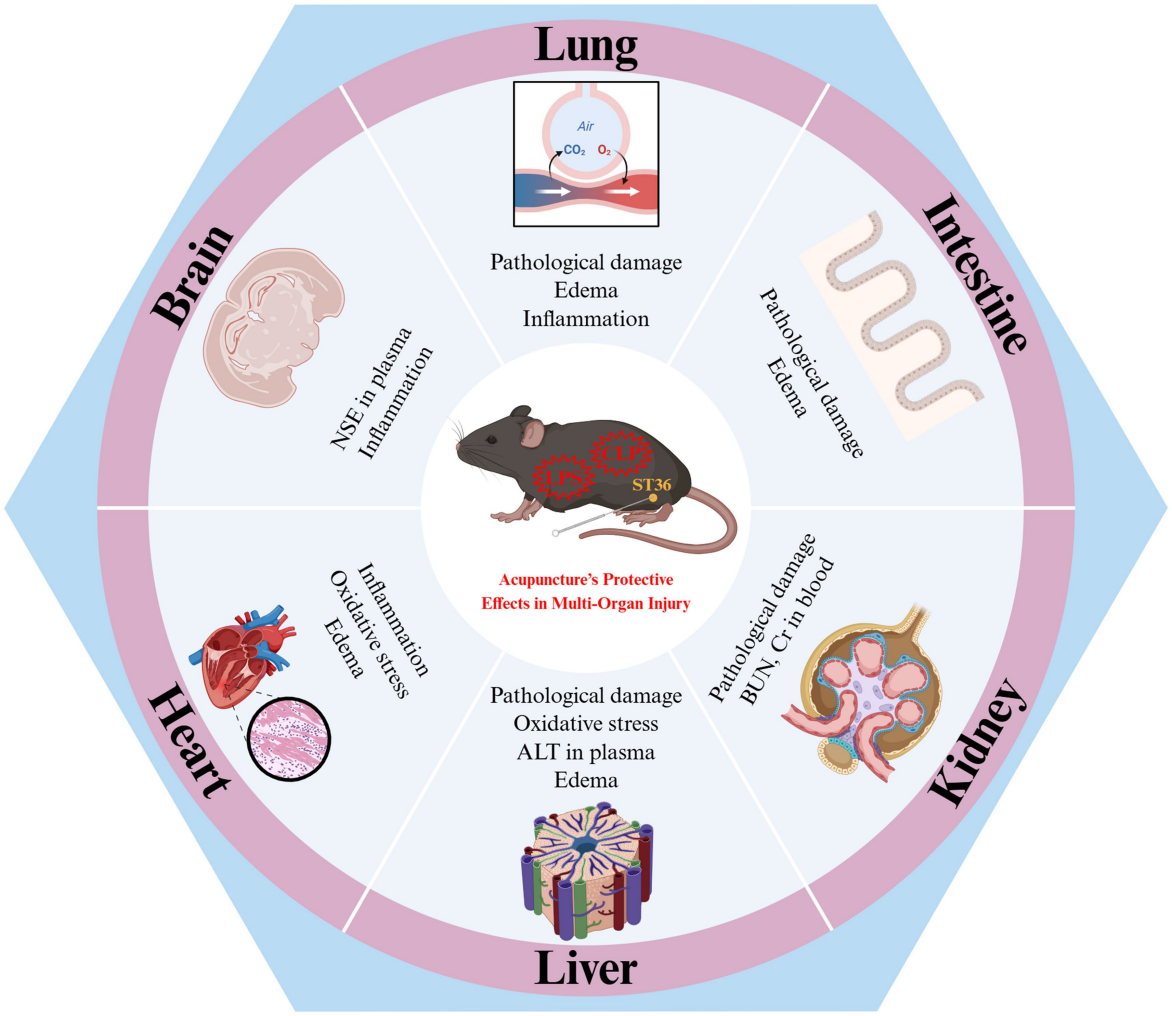
Acupuncture at Zusanli (ST36) for the benefits of sepsis--laboratory evidence for acupuncture’s protective effects in multi-organ injury. ALT, alanine aminotransferase; BUN, blood urea nitrogen; CLP, cecum ligation and perforation surgery; Cr, creatinine; LPS, lipopolysaccharide; NSE, neuron specific enolase; ST36, Zusanli acupoint. Created in BioRender. Zeng, L. https://BioRender.com/0xoe5c2.

A substantial body of research has demonstrated the protective efficacy of acupuncture at ST36 against sepsis-induced organ damage, including lung, bowel, brain, kidney, heart, and liver injuries. The subsequent discussion will first examine the specific damages inflicted on these organs by sepsis and the changes through which acupuncture can mitigate such effects (Figure 1).

#### 4.2.1 Lung

The condition of sepsis is initiated by an imbalance in the immune response within the body. The condition is characterized by an insufficient reduction of inflammation within the body, which increases the risk of damage to multiple organs, with the lungs being particularly vulnerable to sepsis (73). As demonstrated in a series of studies, acupuncture at ST36 has been shown to be effective in alleviating lung injury associated with sepsis. The effectiveness of this treatment has been demonstrated by a reduction in lung injury score (18, 33, 34, 38), lung tissue wet/dry (W/D) weight ratio (33, 37, 38), protein content in bronchoalveolar lavage fluid (BALF) (33, 38), the number of inflammatory cells in BALF (33, 38), and the content of inflammatory factors in BALF (37, 38).

#### 4.2.2 Intestine

Sepsis has been shown to exacerbate sepsis by disrupting the barrier function of the intestinal mucosa, thereby increasing intestinal permeability and leading to bacterial translocation in the intestine. This, in turn, forms a vicious cycle that ultimately drives the development of MODS (74). Recent studies have demonstrated the efficacy of acupuncture at ST36 in alleviating intestinal damage resulting from sepsis. This is primarily evidenced by the attenuation of pathological intestinal damage (26, 27, 34, 35) and the reduction of intestinal tissue water content (26).

#### 4.2.3 Kidney

The development of sepsis is accompanied by a large induced secretion of pro-inflammatory cytokines [tumor necrosis factor (TNF)-alpha and interleukin (IL)-6], which activate the inflammatory process and damage the tissues (75). Prolonged inflammation can lead to a breakdown in tissue perfusion, affecting its normal functioning. For instance, the kidneys are affected by the effects of sepsis (29). The clinical assessment of renal function is performed by laboratory analysis of blood urea nitrogen (BUN) and creatinine (Cr) levels (29). A number of studies have been conducted on the subject, and the results of these studies appear to indicate that acupuncture at ST36 may have a role to play in the prevention and control of sepsis-induced renal injury. The principal findings of these studies suggest that this form of acupuncture can reduce pathological damage to the kidneys (21, 24) and lower the levels of BUN, Cr in the blood (21, 29).

#### 4.2.4 Liver

In the pathogenesis of sepsis, the liver fulfills a dual role: as a site of bacterial and toxin clearance, and as a vulnerable organ in this process (76). The liver is subject to a process of ischemia–reperfusion and oxygen radical damage in response to tissue ischemia and excessive inflammation. Consequently, the effective increase of blood flow to liver tissue, in addition to the reduction of lipid peroxidation and tissue oedema, is imperative for the prevention and treatment of liver injury caused by sepsis (22). In addition, studies have demonstrated the efficacy of acupuncture ST36 in mitigating sepsis-induced liver injury, primarily through the attenuation of hepatic pathological injuries (21, 24), the reduction of hepatic tissue blood flow (22), the diminution of the degree of intrahepatic oxidative stress (21, 22), the decrease in plasma alanine aminotransferase (ALT) activity (22), and the alleviation of hepatic oedema (22).

#### 4.2.5 Heart

During the course of sepsis, there is a tendency for localized tissue ischaemia or ischaemia-reperfusion injury to be triggered by inflammatory overstimulation. This can easily damage the fragile myocardium and lead to cardiac damage (28). Zhang et al. discovered that acupuncture at ST36 can effectively treat and alleviate myocardial injury caused by sepsis. The study found that this treatment primarily reduces the level of CK-MB in plasma, decreases inflammatory factors and oxidative stress in cardiac tissues, and decreases the degree of myocardial oedema (28).

#### 4.2.6 Brain

Sepsis is characterized by the presence of SIRS and MODS. The progression of the disease to a later stage has been shown to cause diffuse brain damage, known as septic encephalopathy (SE). The pathogenesis of SE remains unclear, there is no specific therapy for brain damage, and mortality is significantly increased (77). Wang et al. conducted a research study that yielded findings pertaining to the therapeutic efficacy of acupuncture ST36 in alleviating brain tissue damage caused by sepsis. The study’s findings indicated that this therapeutic effect is primarily achieved by lowering the level of neuron specific enolase (NSE) in plasma and reducing inflammatory factors in brain tissue (23).

In summary, acupuncture at ST36 has been demonstrated to alleviate multi-organ damage caused by laboratory-induced sepsis, with a primary focus on pathological damage to organs, organ oedema, and impaired organ function. The underlying principle that governs this phenomenon can be attributed to the concept of inflammation, which is characterized by the process of cellular activation and the subsequent release of chemical mediators. It has been hypothesized that inflammation is the sole cause of sepsis-related mortality (47). In the context of sepsis, immune homeostasis has been demonstrated to play a crucial role (48). In recent years, there has been a notable shift in the research focus toward the immune activation in sepsis, given the fundamental role of inflammation in the clearance of infections. Consequently, immune stimulation strategies have led to a novel focus in understanding the pathogenesis of sepsis (47).

### 4.3 The molecular mechanisms by which acupuncture protects against multiple organ damage caused by sepsis

Inflammatory imbalance represents the most critical underlying mechanism in the pathogenesis of sepsis, permeating the entire course of the disease (78). In the immune response to sepsis, exogenous factors derived from pathogens (e.g., LPS) and endogenous factors released by injured cells (e.g., high-mobility group box-1 (HMGB-1)) can interact with various PRRs, such as TLRs, NOD-like receptors (NLRs), and C-type lectin receptors (CLRs) (79, 80). The activation of TLRs instigates multiple downstream signaling pathways, with the most prevalent being the mitogen-activated protein kinase (MAPK) and nuclear factor kappa-B (NF-κB) signaling pathways (78), which subsequently result in the production of inflammatory cytokines such as IL-1, IL-6, TNF-*α*, etc. (81). Intracellular pathogens and endogenous danger signals in the cytoplasm bind to NLRs, with NLRP3 participating in the formation of the inflammasome protein complex, releasing cytokines IL-1β and IL-18 to regulate inflammation (82, 83). The association of CLRs with the production of ROS has been well documented, and their role in mediating oxidative stress-induced activation of inflammatory responses has been thoroughly researched (84).

**Figure 2:**
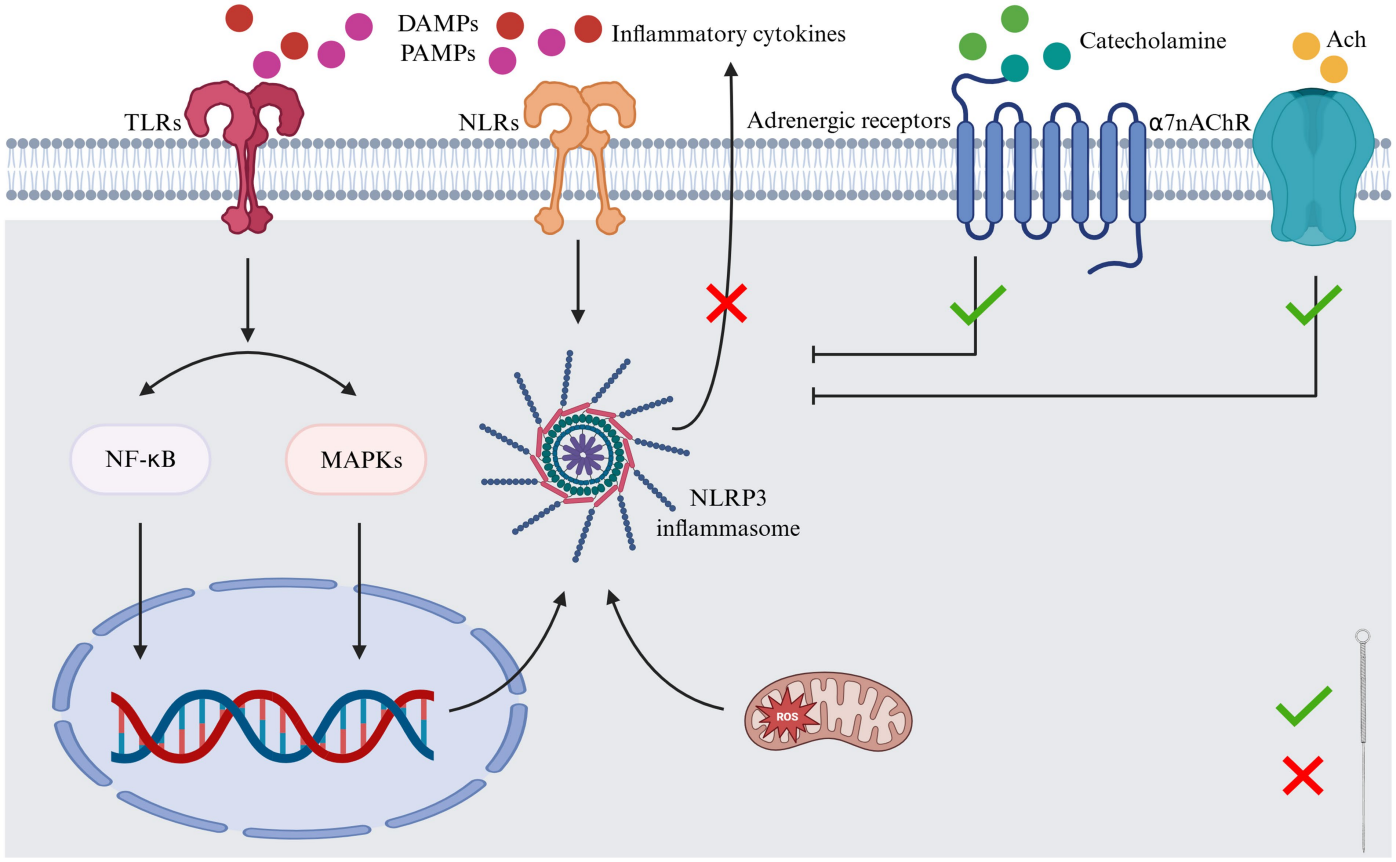
The molecular mechanism of acupuncture at Zusanli (ST36) exerting anti-inflammatory effects. DAMPs, damage-associated molecular patterns; MAPK, mitogen-activated protein kinase; NF-κB, nuclear factor kappa-B; NLRs, NOD-like receptors; PAMPs, pathogen-associated molecular patterns; ST36, Zusanli acupoint; TLRs, toll-like receptors. Created in BioRender. Zeng, L. https://BioRender.com/0i0cln3.

Acupuncture has been identified as an effective modulator of inflammation, with numerous studies demonstrating its ability to regulate inflammatory processes through multiple pathways and mechanisms (Figure 2). These include modulating the MAPK and NF-κB signaling pathways (7), inhibiting oxidative stress via the Nrf2/HO-1 pathway (7), and regulating the NLRP3 inflammasome (85), among others. Acupuncture, defined as a form of mechanical stimulation applied to the skin, has been demonstrated to produce therapeutic effects on target organs. The neuroendocrine-immune system (86, 87) is a key focus of research in this area. A substantial body of fundamental research has demonstrated that acupuncture can modulate the neuroendocrine-immune system (86–88). Research has identified a correlation between homeostasis, dependent on the interaction of the neuro-endocrine-immune network (89), and the regulation of inflammatory activity (90, 91). This finding also suggests the practicality and possibility of using acupuncture to prevent and treat acute inflammatory diseases.

## 5 Acupuncture at Zusanli (ST36) for the benefits of sepsis–laboratory evidence for the “preventive treatment of disease”

The preceding section proffers substantial laboratory evidence to support the ‘holistic’ nature of acupuncture’s anti-inflammatory and immune-modulating effects, and also describes the corresponding molecular mechanisms. However, the mechanism by which the acupuncture effect is transmitted to the target organs to elicit its effects remains to be elucidated. It is fortunate that research has already been initiated on this component of the study, which will further facilitate the unraveling of the mystery of “Preventive Treatment of Disease”.

### 5.1 Anti-inflammatory effects of acupuncture at ST36 through the neuro-endocrine-immune network

In higher animals, the brain has long been considered an immune-privileged organ with a powerful influence on immunity. The immune system, nervous system and endocrine system form a functional regulatory network.

#### 5.1.1 Central nervous system---brain

**Figure 3:**
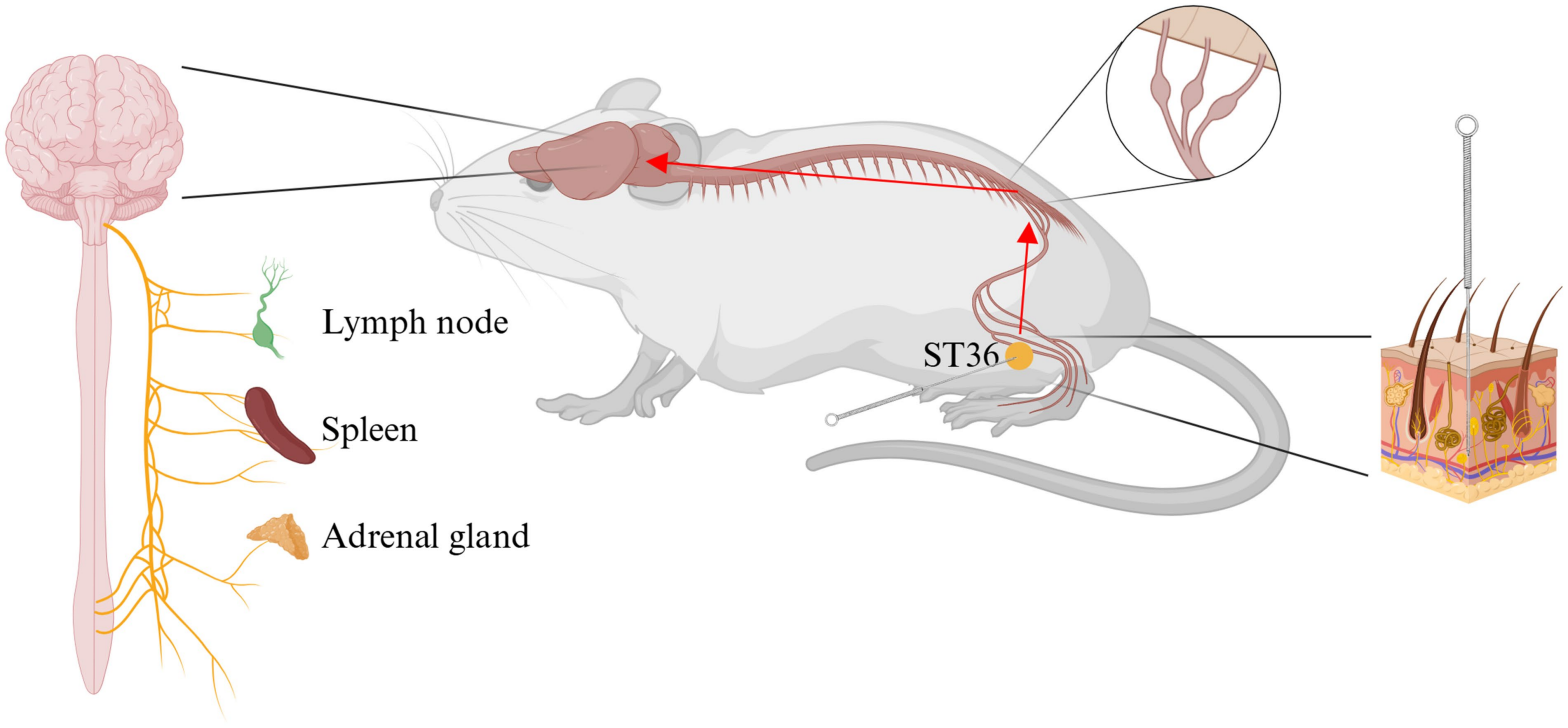
Acupuncture stimulate Zusanli (ST36) exerts anti-inflammatory effects through the neuro-endocrine-immune network. ST36, Zusanli acupoint. Created in BioRender. Zeng, L. https://BioRender.com/f1vu7pc.

The nervous system exerts a significant influence on immune function through two mechanisms: central control and peripheral reflexes (10). These mechanisms are underpinned by neuroendocrine and autonomic nervous control. The impact of psychological stress on immune function serves as a paradigm of central control (92). Peripheral reflex regulation is a more prevalent phenomenon, with the inflammatory reflex being a notable example (93, 94). Inflammatory cytokines have been demonstrated to stimulate peripheral sensory nerves, including both somatic and visceral sensory nerves. In addition, these cytokines have the capacity to enter the brain directly, thereby activating the comprehensive effects of the central nervous system on immune function. The crux of the issue pertains to the central nervous system, specifically, the function of the brain. In order to explore the connection between acupuncture and the brain, brain-computer interface (BCI) systems have been extensively applied in neuroscience research in recent years. The utilization of functional magnetic resonance imaging (fMRI) (95–97) and electroencephalography (EEG) (96, 98–101) has been employed to record and monitor alterations in cerebral activity during acupuncture at ST36. FMRI facilitates real-time, dynamic observation of cerebral functional activity within intact tissue. Consequently, this method can be employed to observe the activation of different brain functional regions following acupuncture treatment (97). The existing literature has demonstrated that scalp EEG has been shown to demonstrate a significant correlation between brain activity and acupuncture stimulation. In order to facilitate more direct observation of the therapeutic effects of acupuncture, a research team designed an electroencephalogram-based monitoring system. The therapeutic impact of acupuncture on the human brain was assessed by extracting periodic and non-periodic characteristics. The findings indicated a substantial augmentation in brain activity within the alpha band (8–12 Hz) during acupuncture sessions, with a notable concentration in the parietal and occipital regions (100). Advances in technology have led to the increased application of deep learning (DL) in the field of EEG analysis. One research team proposed a deep learning framework, the EEG decoder, to establish an acupuncture-brain interface linking somatosensory stimuli with neural representations, while simultaneously revealing effective protocols for assessing the clinical efficacy of acupuncture treatment (102). Another research team proposed the Acupuncture Transformer Detector (ATD), a model based on Convolutional Neural Networks (CNN) and Transformer technology (101). The present study found that acupuncture at ST36 primarily activated the left frontal and parieto-occipital regions. This series of studies provides evidence that lends support to the hypothesis that there is a connection between acupuncture and the brain. The effects of acupuncture are theorized to be mediated through the brain’s activation of the neuro-endocrine-immune network, ultimately realized via outputs from the neuroendocrine or autonomic nervous systems. The neuroendocrine output is primarily involved in the regulation of immune activity via the hypothalamic–pituitary–adrenal (HPA) axis, the hypothalamic–pituitary-thyroid (HPT) axis, the hypothalamic–pituitary-gonadal (HPG) axis, and the hypothalamic-growth-hormone (HGH) axis (103). The autonomic nervous system (ANS) comprises the sympathetic and vagus nerves, both of which regulate the immune system and inflammation (10), a subject which will be the primary focus of this article (Figure 3).

#### 5.1.2 Peripheral nervous system---autonomic nervous system

##### 5.1.2.1 Sympathetic nervous system

The sympathetic nervous system contains nerve fibers (from postganglionic neurons) that specifically innervate immune organs, including primary lymphoid organs, namely the bone marrow and thymus, and secondary lymphoid organs, namely the spleen, lymph nodes, and mucosa-associated lymphoid tissue (104, 105). Postganglionic sympathetic neurons have been shown to release the neurotransmitter norepinephrine, which activates *β*- and *α*-adrenergic receptors on immune cells, thereby producing regulatory effects (10, 106). Following a substantial number of experiments, it has been determined that the greater splanchnic sympathetic nerves are the sympathetic nerve efferent arm of the inflammatory reflex. These nerves have been shown to inhibit the release of inflammatory cytokines in the spleen and other organs innervated by these nerves, which are located internally (107–109). It is hypothesized that this phenomenon may be associated with the effective regulation of microRNAs (miRNAs) in T cells within the spleen (110). Concurrently, given the recognized function of somatic sympathetic nerve reflexes, selective stimulation of somatic nerves connected to the same spinal cord segment as sympathetic nerve efferent fibers that innervate specific organs or tissues can exert an immunoregulatory effect on local specific organs or tissues (30). A further study established that an EA of 3.0 mA administered to the ST36 acupoint on the hindlimb prior to the injection of LPS can also activate the spinal sympathetic axis and produce vagal nerve output, independent of the effects of anti-inflammatory drugs (30). In a similar vein, Kim’s team demonstrated that EA at ST36 can inhibit the occurrence of peripheral inflammation by activating postganglionic sympathetic neurons (111, 112).

##### 5.1.2.2 Vagus nervous system

The vagus nerve, classified as the tenth pair of cerebral nerves, has been determined to comprise 75% of the body’s parasympathetic fibers (15). First, it can receive inflammatory stimulation (113–115); second, it can receive acupuncture stimulation (32). Of particular significance is its capacity to modulate the inflammatory response, a function that has been demonstrated in numerous studies (116). These studies have demonstrated that direct stimulation of the vagus nerve can elicit a substantial anti-inflammatory effect. A substantial body of research has indicated that the vagus nerve has the capacity to regulate inflammation through the spleen (117–119). However, a recent study has revealed an absence of a neural connection between the spleen and the vagus nerve (120). Nevertheless, the relationship between the vagus nerve and anti-inflammation is well-established, and a substantial body of new studies has emerged that demonstrates the vagus nerve’s role in modulating inflammatory response pathways (32, 121, 122). While acupuncture at ST36 has been demonstrated to stimulate the vagus nerve (10), the anti-inflammatory effects of the nerve are conveyed through both direct and indirect pathways.

###### 5.1.2.2.1 Cholinergic anti-inflammatory pathway--- direct effect

Efferent vagus nerves have been demonstrated to impede the secretion of pro-inflammatory cytokines and forestall systemic inflammation. This vagal function has been termed the “cholinergic anti-inflammatory pathway” (93, 123). The pulmonary parasympathetic inflammatory reflex, for instance, comprises three primary components: the vagus nerve sensory (visceral) neurons, which are located in the distal airways or alveoli; the nucleus tractus solitarius (NTS) information integration center in the brainstem; and the distal pulmonary epithelium, which innervates the epithelial cells on the vagus nerve efferent (motor) cholinergic fibers (115). The transmission of signals from acupuncture at ST36 into the NTS (124, 125) has been demonstrated. When a specific level of signal input is provided to the NTS, the subsequent signaling outflows to the vagal nerve endings at the alveoli. These endings synthesize and release Acetylcholine (Ach), activate the alpha 7 subunit nicotinic acetylcholine receptor (α7nAChR) on the surface of nearby cells, and ultimately serve to modulate the inflammatory response in the lungs (33, 115). In addition to the lungs, vagal efferent cholinergic fibers project to numerous internal organs, including the heart, liver, gastrointestinal tract, kidneys, and pancreas (126). This anatomical basis underlies the therapeutic efficacy of acupuncture at ST36 in reducing sepsis-induced organ inflammation. In relation to this pivotal mechanism, a substantial number of laboratory articles have been published on the effects of acupuncture ST36 on sepsis-induced organ damage. Employing vagotomy to perform a protective effect/therapeutic effect flip serves to corroborate the validity of this pivotal mechanism (22, 24, 28, 30, 33). Furthermore, the study’s findings are corroborated by additional research, which utilizes inhibitors or agonists of α7nAchR to demonstrate this mechanism (31, 33, 38). This finding also elucidates the mechanism by which acupuncture exerts a therapeutic effect on inflammatory diseases. However, the precise mechanism by which acupuncture preconditioning produces these effects remains to be elucidated.

###### 5.1.2.2.2 Vagal- splanchnic nerve axis--- indirect effect

In addition to the previously mentioned direct anti-inflammatory effect of acupuncture at ST36 via vagus nerve-mediated cholinergic anti-inflammatory pathways localized to multiple organs, further research is needed to determine the specific mechanisms by which acupuncture at ST36 exerts its anti-inflammatory effects. It has been found that acupuncture (blue-light stimulation) at ST36 exerts anti-inflammatory effects indirectly through the sciatic nerve-mediated vagus-adrenal axis (127) by prompting the secretion of dopamine and epinephrine into the bloodstream from the adrenal glands (32, 72). Additionally, it has been demonstrated that efferent fibers of the vagus nerve can transmit signals to the splanchnic nerve via the celiac ganglia and the superior mesenteric ganglion (128, 129). This results in an increase in the level of intra-splenic Ach (119), which, in turn, leads to the inhibition of the release of TNF and other pro-inflammatory cytokines. Research has demonstrated the role of the vagus nerve in mediating immune response and inflammatory regulation through the splanchnic nerve (130–132). Acupuncture at ST36 has been demonstrated to regulate sepsis via T lymphocytes (31, 34). The relationship between the vagus nerve, the spleen, and this regulatory process merits further investigation. The potential of this sequence of actions to serve as the foundation for the anti-inflammatory effect of acupuncture preconditioning/pretreatment warrants further investigation.

In summary, it is evident that acupuncture at ST36 can elicit autonomic nervous system immune reflexes by means of stimulation of the surrounding somatic nerves, predominantly through sympathetic nervous system immune reflexes and vagus nerve immune reflexes (133, 134). This provides a theoretical and fundamental basis for the application of acupuncture in acute inflammatory conditions. Acupuncture also has widespread clinical applications, such as in the treatment of the most severe infectious disease in recent years, Coronavirus disease 2019 (Covid-19), where it can be used to improve various clinical and associated symptoms (135). Furthermore, the practice of acupuncture has been demonstrated to facilitate the establishment of connections with the autonomic nervous system, immune organs and adrenal glands, resulting in the secretion of neurotransmitters and hormones. It has been demonstrated that the drug can target adrenergic receptors located on the surfaces of innate immune cells throughout the body, thereby establishing a local neuroimmune communication system that assists in regulating ongoing immune responses (136, 137). This finding provides compelling evidence for the delayed effects of acupuncture preconditioning/pretreatment.

### 5.2 Anti-inflammatory effects of acupuncture at ST36 through the exosomes

The preceding section concentrated on providing a synopsis of the manner in which acupuncture at ST36 can stimulate the surrounding somatic nerves, thereby inducing an autonomic nervous system immune reflex. Furthermore, the study demonstrated that acupuncture at ST36 can interact with organs such as the spleen and adrenal glands to produce catecholamine substances. These substances act on adrenergic receptors located on the surface of immune cells, thereby inducing immune regulatory functions. Moreover, an additional pivotal ‘communication particle’ between multiple organs has been identified as exosome. Exosomes are defined as nanoscale lipid membrane-encapsulated particles that are derived from almost all types of cells present in many body fluids. The primary function of the substances in question is to protect the cargo from the process of enzymatic degradation in body fluids (138), thereby ensuring the stability of the materials they contain. It has also been demonstrated that exosome transfer cargo between different locations within cells or within the body, thereby mediating intercellular communication under physiological and pathological conditions (139). To articulate the matter differently: exosome possession of inherent advantages facilitates their efficient delivery of proteins and genes to target cells (140), thereby contributing to a variety of biological processes (141). The sources of serum exosome are also extremely diverse, including autonomic nerves such as the vagus nerve (142) and sympathetic nerve (143), immune organs such as the spleen (144) and lymph nodes (145), and immune cells such as T cells and so on (139, 146–148). Acupuncture, a form of alternative medicine, has been shown to have a comprehensive influence on the aforementioned factors. This influence has been the subject of research by some teams. The extraction of the sample from the serum is conducted using the Exo Quick Precipitation method, and subsequent identification and confirmation of the sample as an exosome is performed (18, 19). Subsequently, intraperitoneal injection of serum exosome in ST36 mice with electroacupuncture demonstrated anti-inflammatory effects on sepsis-related organ damage, which was predominantly associated with the microRNAs carried in serum exosome (18). However, the efficacy of acupuncture in sepsis has been shown to be reversed by exosome antagonists (18). Consequently, the group advanced the concept of “Acupuncture network drug,” proposing that the serum exosome secreted by the body following acupuncture intervention could be employed in the development of acupuncture network drugs with low immunogenicity, which may offer significant advantages in the domains of drug development and modification (149). This study explores an alternative material basis for the conduction of the acupuncture effect and provides research ideas for the generation of the acupuncture preconditioning effect. Additionally, a close correlation has been observed between the presence of exosome-like particles and the occurrence of sepsis, both in terms of therapeutic interventions (150) and prognostic implications (151). Consequently, there is an urgent need for further research to identify the cell of origin of these exosome formations, particularly those induced by acupuncture at ST36, and to pursue more in-depth studies in this area. This provides a stronger theoretical and fundamental basis for the use of acupuncture in the treatment of disease prevention. Furthermore, it has been hypothesized that exosome influence on the central nervous system may be a consequence of their capacity to activate afferent vagus nerve pathways into the brain (152, 153). In addition, the production and dissemination of exosome has been demonstrated to act as an intermediary connection between the central nervous system and the peripheral nervous system (154). Further investigation into the relationship between exosome and the neuro-endocrine-immune network may prove to be a fruitful avenue for future research endeavors.

## 6 Discussion

**Figure 4:**
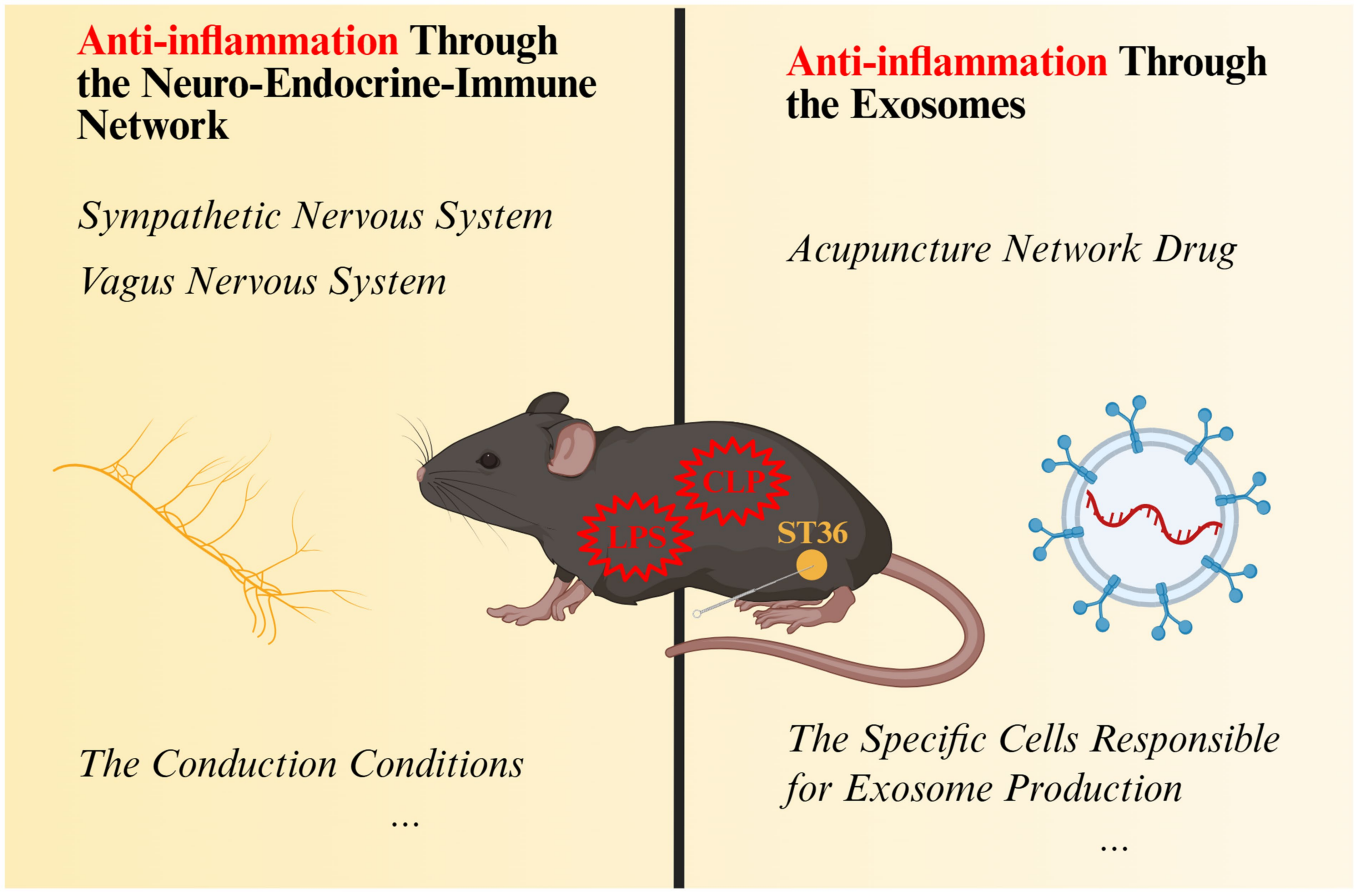
How does acupuncture stimulate Zusanli (ST36) play a systemic anti-inflammatory effect. CLP, cecum ligation and perforation surgery; LPS, lipopolysaccharide; ST36, Zusanli acupoint. Created in BioRender. Zeng, L. https://BioRender.com/08874wi.

The present article does not concentrate on describing the signaling pathways through which acupuncture at ST36 exerts its effects; rather, it endeavors to elucidate how acupuncture at ST36 as a whole exerts an anti-inflammatory effect on the inflammatory damage of multiple organs in sepsis. According to the current state of research, stimulation of ST36 by acupuncture can mediate the secretion of hormones from the neurocircuitry as well as the promotion of exocytosis produced by cells in the organism to achieve the modulation of inflammation (Figure 4). This finding lends further credence to the ‘Preventive Treatment of Disease’ and ‘Holistic Concept’ postulated within the framework of traditional Chinese acupuncture theory, thereby underscoring the existence of a tangible, material foundation for these concepts. Concurrently, substantial clinical evidence indicates that acupuncture can be employed for the prevention and treatment of obesity (155), urticaria (156), Alzheimer’s disease (AD) (157), and Coronavirus disease 2019 (Covid-19) (135), among numerous other acute or chronic inflammatory or neurodegenerative conditions. A substantial body of research has been dedicated to the exploration of obesity-related comorbidities (158), with a predominant focus on acupuncture’s potential in regulating neural circuits and the endocrine system. Nevertheless, there are as yet unresolved issues in fundamental experimental work. First, in terms of neural circuits, although there is an important discovery on the effect conduction of acupuncture at ST36 (32), which initially lays the anatomical foundation for the effect neural conduction of acupuncture. However, it is imperative to acknowledge that the organism functions as a coordinated entity, and the conduction of acupuncture at ST36 does not occur exclusively through the vagus-adrenal axis. Instead, it is also influenced by spinal sympathetic reflexes (30, 32). Consequently, future research should explore a range of factors, such as the conduction conditions-electroacupuncture parameter settings, that modulate the diverse neural circuits involved in treating specific diseases. Moreover, the incorporation of neuroanatomical tracing (159) or optogenetic validation (160) in animal models would serve to substantiate mechanistic assertions. Secondly, in the context of differentially expressed microRNAs (exosomes), although it has been tentatively concluded that serum exosome production in ST36 normal mice induced by acupuncture has anti-inflammatory effects (18), further research is necessary to determine the precise mechanisms underlying exosome production, identify the specific cells responsible for exosome production, and ascertain the most effective exosome targets. Finally, the majority of research is currently centered on the regulation of inflammation after its onset. Observations have revealed that low-intensity electroacupuncture stimulation at ST36 in the hindlimb activates the vagal-adrenal anti-inflammatory pathway in a manner that is independent of disease state, thereby offering an alternative approach to the treatment of predefined systemic inflammation (30). Our research group’s previous findings indicate that electroacupuncture pretreatment exerts a significant protective effect on LPS-induced acute lung injury (ALI) in mice (161). Concurrently, serum exosome production by acupuncture at ST36 normal mice exhibited anti-inflammatory effects (18). These findings lay the foundation for research initiatives that explore the potential of acupuncture in disease prevention. However, it is imperative to integrate the inquiries raised in the preceding two points to facilitate more profound investigation. For instance, conducting in-depth investigations into how variations in electroacupuncture parameters influence distinct stimulation of the sympathetic and vagus nerves, alongside the resulting differences in secreted exosome, and further exploring the neural circuitry linking these effects to acupuncture mechanisms. Concurrently, the incorporation of sophisticated experimental methodologies, including neuroimaging techniques (e.g., fMRI for brain organ crosstalk) or exosome and single nucleus RNA sequencing (for the purpose of simultaneously sequencing the single-cell transcriptome and exosome microRNA in the same tissue), can further address the unresolved issues raised in the preceding text.

## Glossary

A: adrenaline
AAV: adeno-associated virus expressing
Ach: Acetylcholine
AD: Alzheimer’s disease
ALT: alanine aminotransferase
α7nAChR: alpha 7 subunit nicotinic acetylcholine receptor
ANS: autonomic nervous system
AST: aspartate aminotransferase
ATD: Acupuncture Transformer Detector
BALF: bronchoalveolar lavage fluid
BCI: brain-computer interface
BUN: blood urea nitrogen
CARS: compensatory anti-inflammatory response syndrome
CK-MB: creatine kinase MB isoenzyme
CLP: cecum ligation and perforation surgery
CLRs: C-type lectin receptors
CNN: Convolutional Neural Networks
Covid-19: Coronavirus disease 2019
Cr: creatinine
DA: dopamine
DIC: disseminated intravascular coagulation
DL: deep learning
ECs: endothelial cells
EEG: electroencephalography
DAMPs: damage-associated molecular patterns
DAO: diamine oxidase
GSH-Px: Glutathione peroxidase
HGH: hypothalamic-growth-hormone axis
HMGB-1: high-mobility group box-1
HPA: hypothalamic–pituitary–adrenal axis
HPG: hypothalamic–pituitary-gonadal axis
HPT: hypothalamic–pituitary-thyroidaxis
IFN: Interferon
IL: interleukin
LPS: lipopolysaccharide
MAPK: mitogen-activated protein kinase
MDA: Malondialdehyde
miRNAs: microRNAs
MODS: multiple organ dysfunction syndrome
MPO: myeloperoxidase
NA: noradrenaline
NF-κB: nuclear factor kappa-B
NIH: National Institutes of Health
NK: natural killer
NLRs: NOD-like receptors
NO: nitric oxide
NSE: neuron specific enolase
NTS: nucleus tractus solitarius
PAMPs: pathogen-associated molecular patterns
PRRs: pattern recognition receptors
ROS: reactive oxygen species
SE: septic encephalopathy
SIRS: systemic inflammatory response syndrome
SOD: superoxide dismutase
ST36: Zusanli acupoint
TCM: traditional Chinese medicine
TCRs: T cell receptors
TGF: transforming growth factor
TLRs: toll-like receptors
TNF: tumor necrosis factor
W/D: wet/dry
WHO: World Health Organization.
